# Physiology

**Published:** 1868-02-15

**Authors:** 


### CHICAGO MEDICAL JOURNAL.

*Vol. XXV.—FEBRUARY* 15, 1868.—*No.* 4.

### PHYSIOLOGY.

*Investigations into the nature of Miasmata furnished by the human body in health (read before the Academy of Sciences, the* 10*th of September*, 1867, *by Dr. Lemaire J. Paris*).

TRANSLATED EXPRESSLY FOR THE JOURNAL BY WALTER HAY, M.D.

For a very long time, experience has taught physicians and veterinary surgeons that both men and animals, in health, assembled in large numbers in a confined atmosphere, speedily communicate to it new properties, which engender transmissible maladies. It is under these circumstances that the typhus of prisons, ships and ambulances, typhoid fever, as well as the typhus of animals, glanders, etc.

Physicians, from the time of Galen up to the commencement of this century, impressed with the peculiar appearances presented by the fluids and tissues in these diseases, referred their alteration to putridity. Their opinion upon this point was so firmly established, that they gave the names *putrid degeneration* and *hospital gangrene* to the special transformations sustained by wounds in this confined air, and of *putrid fever* to typhus and other maladies which presented analogous characteristics.

It must be admitted that there were opponents to this mode of considering these phenomena. A certain number of physicians could not admit that the fluids and solids could undergo putrefaction during life.

At this epoch, the term *putrid fever* is abandoned to history, and erased from medical nomenclature.

At those periods, the causes of putrefaction were unknown; it was only by analogy that physicians admitted the putridity of the fluids. Hence, their opinions were only based upon an hypothesis which other hypotheses have subverted.

In order to elucidate this subject, I have undertaken some experiments, the results of which, appear to me to demonstrate that the morbid phenomena, attributed to putridity by the older physicians, are the work of microzoa, which determine the putrefaction of organic matters.

Before detailing my experiments, I beg the Academy to permit me, by reason of the importance of this subject, to review rapidly the state of science upon this point.

The frightful ravages occasioned amongst men and animals by the air of confined localities, have, for a long time, stimulated the zeal of a great number of very distinguished physicians and chemists, who have undertaken researches in the hope of discovering their causes.

By applying the terms miasma, effluvium, to the supposed causes of these maladies, physiologists have learned nothing of their nature; although it must be acknowledged that they concede and designate something to be sought—to be discovered. The same may be said of the numerous and diverse theories which have been imagined. These names and these theories propose a problem without solving it, and one too which still awaits solution.

The physicians of the 16th, 17th, 18th, and 19th centuries had recourse to the idea of a ferment to explain the causes of the alterations which I have pointed out. But what diversities of opinion do these words ferment, and fermentation embrace? Some, seeing only motion and heat produced by fermentation, make use of them to explain the contraction of the ventricles of the heart; the heat, the agitation and the acceleration of the pulse in fever; others consider the secretion of the saliva, of bile, of semen, etc., as the products of fermentation, etc., etc. A learned Jesuit, Athanasius Kircher, foresaw the nature of ferments. In his opinion it was to the animalculæ, the worms and the insects which are found in matters in process of alteration, that fermentation was to be attributed. That their eggs are the leaven of ferment, which gives origin to this phenomenon.

The propagation of transmissible maladies, was the work of these animalculæ, these worms, these insects. This theory had numerous partizans during more than a century, after which it was completely abandoned.

M. Raspail, who has attempted to revive it, has only attracted the sarcasm of physicians. The author of the theory, equally with M. Raspail, lacked evidence sufficient to cause it to be accepted definitely. The imagination played too prominent a part in it. With others, the ferments which produced diseases were leavens of a peculiar nature. In our time a ferment has been considered both as a vegeto-animal matter, and as an albumenoid matter altered by oxygen.

In presence of such diverse opinions upon the nature of ferments, it was impossible to understand them, and to recognize the true cause of the maladies attributed to ferments, so the question remained without solution.

The brilliant researches of M. Cagnard-Latour upon alcoholic fermentation, the experiments of Schultz and Schwann, and other savans, again attracted attention toward living ferments. I believe that I assisted in demonstrating, in my investigations upon coal-tar and phenic acid, that microphytes and microzoa produce the fermentation termed spontaneous. This investigation has thus entered upon a new field, but a different one from that which Kircher traced, and which M. Raspail has attempted to revive.

Chemists of the first order have endeavored to find the solution of the problem of the nature of miasmata by analyzing the air. Their experiments upon confined air demonstrate that its composition differs from that of pure atmospheric air, by a much greater quantity of watery vapor, by much carbonic acid, by traces of sulphuretted, ammoniacal and carburetted hydrogen gases, and finally, by a larger quantity of organic matters. The experiments of Messrs. Regnault and Reiset upon respiration, which were made with great precision, demonstrate that warm-blooded animals disengage by perspiration only indeterminable quantities of sulphuretted and ammoniacal gases. It is also in almost unappreciable quantities that the presence of carburetted hydrogen has been determined. It can not be, then, that the diseases above referred to can be reasonably attributed to these last named bodies, since there exist of them only harmless traces.

It remains, then, to examine the influence of the carbonic acid, the watery vapor, and the organic matters. It is very evident, that the carbonic acid exhaled by the lungs, accumulates in confined places, which may thus become irrespirable and very dangerous from deficiency of oxygen, if there does not exist sufficient communication with the external air to permit its renewal. The increase in the amount of the watery vapor may impede respiration. These facts are well known, and therefore I will not dwell upon them longer.

The results furnished by chemical analysis have been deemed insufficient, not only to explain the symptoms which the patients present, the nature of the alterations which the fluids and the solids exhibit under these circumstances, but even to account for the mode of transmission, of the propagation of these maladies, and the immunity which certain individuals enjoy.

For example, how a man or an animal, not sick, or merchandize going out of a vessel or a stable where typhus prevailed, could transport the cause of this disease thousands of miles, and propagate it in a ratio sometimes very considerable.

Since thirty years ago, a very prominent function has been assigned to the organic matters contained in the air.

In the investigations upon ferments and miasmata, which extend back to 1859, I was compelled to determine that the essential characteristic of transmissible maladies (contagions of authors), is the reproduction and the multiplication of the specific morbid element (morbid species), that the cause of these diseases can not, in my opinion, be attributed to chemical bodies, nor to organic matters, because the former, no more than the latter, can reproduce or multiply themselves. For example, an animal is poisoned by rattlesnake virus, by curare, by arseniated hydrogen, by strychnia, or by any other poison, solid, liquid, or gaseous, this animal can not transmit the cause of its disease or death by contact, or by the interposition of the air to other animals of its kind. The action of these poisons is limited to individuals, because they can not reproduce or multiply themselves.

In numerous cases of poisoning, occasioned by the virus of serpents, by mineral, or by vegetable poisons, I do not think that it has ever been asserted, that the subjects who have died after the inoculation of the virus, or the ingestion, or the inspiration of the poisons, have transmitted the cause of their disease or their death, by contact, or by the exhalations from their bodies, to friends who have visited them, to relatives who have nursed them, nor to those who have watched, buried, or exhumed them; whilst miasmata transmit themselves under all these circumstances, originate the diseases of which I have spoken, and multiply themselves.

This is not all; poisons—solid, liquid, or gaseous—produce their deleterious effects, so to speak, instantaneously. Miasmata, on the contrary, have a period of incubation and of development, exhibiting themselves by different symptoms, called by physicians premonitory (prodromata). They may continue several days before threatening the life of the individual. Finally, if it is attempted to explain the propagation and the multiplication of miasmatic diseases from Europe to America, or to Africa, or reciprocally from those countries to Europe by chemico-gaseous poisons, the only ones whose existence can reasonably be supposed, this explanation appears to me impossible.

Indeed, how can it be supposed, that a small quantity of gaseous poison can be transported across seas without injury to the person bearing it, resist evaporation, produce among other individuals new symptoms of poison, and propagate them to a whole population?

Thus, whatever may be the action of a chemical poison, it must be admitted that it is impossible to explain, by means of it, the reproduction and multiplication of the morbid species, not only in a large number of inhabitants of a city or of a nation, but in those of an entire world, as is apparent in the case of variola, cholera, typhus, etc.

It is, in my opinion then, necessary to seek elsewhere than in the chemical compounds the solution of this difficult problem. Experts of great skill, having found nothing by chemical analysis, in seasons of epidemics, in the atmospheric air, which could be referred to miasmata, have denied their existence.

M. Chevreul, in a learned report which he read to the Academy in 1839, upon an epizootic which raged amongst the cattle of Paris and its environs, said, that in denying the existence of miasmata, because it had not been demonstrated by chemical analysis, they had gone too far. He foresaw the possibility of making this demonstration by the aid of mechanical or physical means, by compression or by cold. It is precisely by the aid of cold, that I have, since 1861, demonstrated the nature of putrid miasmata, and three years later, we demonstrated, by the same means, with my excellent friend Prof. Gratiolet, that the air of the most unhealthy portion of Sologne, contained also microphytes and microzoa in process of development.

The experiments which I shall have the honor to submit to the Academy, have also been made, by the aid of cold and of the microscope, upon the vapor of water contained in the atmosphere of localities which I will indicate hereafter.

The first difficulty to be surmounted, in these investigations, has been the choice of subjects. I have considered that soldiers in active service, in the prime of life, subjected in time of peace to a systematic mode of life, to a wholesome alimentary regimen, uniform for all, would afford me all desirable guarantees of men in a perfect state of health. Thanks to my friend M. Reiron, Capt. and Adjt. of the 4th Regt. of Voltigeurs of the Imperial Guard, I have been enabled to make experiments upon the air of the chamber occupied by the men of his company, barracked at Fort de l'Est, near St. Denis. These soldiers had returned some days since from the very healthy camp at Chalons.

The Fort de l'Est, separated from every habitation, overlooks the plain of Aubervilliers. It is in good sanitary condition.

My experiments were made on the 19th of September, 1866, from four to half-past five in the morning, simultaneously at three points—that is, 1st, upon the air of a chamber of the barrack; 2nd, upon that of a casemate; and 3rd, upon the external air; this last to serve as a standard of comparison. I chose this early hour in the morning to experiment, as the soldiers were yet in bed, the doors and the windows shut, and also because profound quiet reigned upon the exterior of the fort. By these precautions, I avoided the causes of agitation of the air, which would have attracted different sorts of dust, and rendered my experiments less conclusive.

The Voltigeurs had been in bed since nine o'clock in the evening, hence the air was loaded during more than eight hours with exhalations from their bodies.

### EXPERIMENT I.

Made in the barrack of the fort, on the second floor, in a chamber containing twenty-four beds, of which twenty were occupied. Its cubic content is about 420 metres. Its walls and ceiling had been white-washed with lime. It is provided with two large windows, one to the east, opening upon the plain, the other to the west, upon the large court-yard of the fort, and a large door, closing the chamber upon the interior stairway, are the only openings which it possesses. There is no chimney nor ventilator. The temperature of this room, at the moment when the experiment was made, was 18° Centigrade (70°·16 Fahr.) The odor of its atmosphere, *sui generis*, was disagreeable. I collected in the middle of it, by the aid of my apparatus filled with ice, about six grammes of watery vapor reduced to a liquid; I placed this in a new flask, of sixty grammes capacity, previously washed with distilled water, and closed it with a new cork, likewise washed.

At the moment of its condensation, the liquid was colorless and limpid, its odor being the same as that which I had perceived in the atmosphere of the room. Its taste was slightly pungent. It exercised no appreciable influence upon blue or red litmus paper, nor upon those of turmeric or lead.

The microscopic examination was made two hours after the condensation, in order to give the liquid time to place itself in equilibrium with the atmosphere, my object being to ascertain if there existed microphytes, or microzoa, it was necessary to give it time to become warm again, because at zero animalculæ give no sign of life. The thermometer indicated 16° Centigrade. At this period of the experiment, the microscope enabled me to determine, independent of few organic fragments and grains of dust, the existence of a considerable number of diaphanous bodies, whose forms could be referred to the following: spherical, ovoidal, cylindrical, regular, and irregular. The cylindrical bodies were about one to two thousandths of a millimetre in width, and three thousandths of a millimetre in length. The diameter of the sperical and ovoidal bodies, varied from fifteen to twenty ten thousandths of a millimetre. These bodies, as we shall see, are microphytes and microzoa in process of development. A new examination, made four hours after the preceding one—that is to say, six hours after the condensation—gave me the following results: The diaphanous bodies, of which I have just spoken, were much more numerous than at the first examination. They existed by thousands in a little drop of the liquid. Moreover, bacteria termo and puncta were in motion; little wand-like vibriones executed their rapid undulatory movements. Independently of these little beings, I found a very large number of a species of animalcules, whose existence I had not yet established in my microscopic investigations. I will describe them. They are diaphanous, ovoid; do not present any appreciable opening or filaments. They execute movements rapid in every sense. The greater number presented, in the middle portion, a very decided circular depression, which appeared to mark the place of division for their reproduction. The dimensions of an individual did not present much variation from fifteen to twenty ten thousandths of a millimetre in length, and from ten to fifteen ten thousandths of a millimetre in breadth. Is it a vibrio? If their simple organization alone, it is not incredible. But if, with Ehrenberg and Dujardin only, those animalcules are to be considered vibriones, whose bodies are flexuous in their movements, these can not be classed amongst vibriones, since they do not present this characteristic.

The monads, whose organization are most simple, have a flagelliform filament. I have not been able to determine the existence of this filament. I have, however, an excellent microscope, and I work with a magnifying power of six hundred diameters.

Ehrenberg described oviform monads, some indented, others not. These characteristics are very nearly those presented by the animalculæ just described. Dujardin, who criticises very severely the works of Ehrenberg, says that he has never seen these any more than other species of monads described by the learned Prussian micrograph, and appears to doubt their existence. In spite of the consummate skill of Dujardin, I must say that the characteristics of this animalcule appears to me to associate it in all its points with the ovoid indented monads of Ehrenberg. Could this animalcule be considered the cause of typhus? I will discuss this question hereafter. Before resuming the description of my experiments, I beg the Academy to remark carefully that, six hours after the condensation of the watery vapor, I found in this liquid two species of bacteria, wand-like vibriones, and the ovoid monads of Ehrenberg in great numbers, all executing their habitual movements. This fact is all the more interesting, because there are never found in so short a time, so great numbers of animalculæ in the vapor of atmospheric water collected in healthy localities, nor even in infusions prepared with fresh matters. There is necessary, at least, a period of forty-eight hours, and a temperature of 16° Centigrade, to determine their existence. Moreover, it may happen that no animalculæ are developed in the vapor of water collected in the external air. I have made M. Chevreul a witness of a fact of this kind, in an experiment made under his eyes.

It must necessarily be that the animalculæ, of which I had just spoken, had commenced to develop themselves to some extent. When I shall have demonstrated their existence in considerable quantities upon the human body, this fact will be easily explained. I return to my experiments.

Twenty-four hours after the condensation, I found, in the liquid already referred to—that is to say, in a single drop of it—numerous bacteria termo; some isolated, others agglomerated, forming little masses composed of ten, twenty, or even a hundred of these animalculæ, a few bacteria, catenula, and puncta; numerous wand-like vibriones very active, a great number of ovoid monads; finally, spores, ovoidal, and spherical of from fifteen to thirty-five ten thousandths of a millimetre diameter. The little ovoid, spherical, and cylindrical bodies, which were so numerous in the first hours, had diminished in considerable proportion. The fact, which confirms some others I have already published, authorizes me, still more strongly, to assert that their number is in the inverse ratio to that of the animalcules and the spores; considerable at the beginning of the experiments, it diminished in proportion as that of the animalculæ and spores increased. Is not this proof that these little bodies are infusoria in a rudimentary state, whose germs authors admit to exist, without having seen them?

EXPERIMENT II.

Made upon the confined air of a casemate. It contained thirty-eight beds, of which seventeen were occupied, the cubic content being 530 metres. Its floor is bitumen, and its walls, rough-cast to the height, had been recently white-washed with lime. A little window (embrasure) opens upon the moat, which only contains a little rivulet in the centre. A large window-frame, which occupies all the height and width of the casement, closes it upon the great interior court-yard of the fort.

I expected to find the air of this casemate much loaded with miasmata, but my anticipations were not realized. I was struck, upon entering, with the difference which existed between the odor of its atmosphere and that of the chamber of the barrack already referred to. I suspected, therefore, that it was provided with ventilators, whilst that of the chamber of the barrack, as has been stated, was deprived of this excellent means of purification. M. Reiron, indeed, questioned the chief of the chamber, who showed us two ventilating tubes placed in the ceiling.

I will state, that the number of soldiers in proportion to that of the beds, was much smaller, since in the chamber of the barrack twenty out of twenty-four were occupied, whilst here, but seventeen out of thirty-eight were tenanted. This conditioned, in addition to the presence of the ventilators, ought to contribute to render its atmosphere less vitiated.

The watery vapor was condensed in the middle of the casemate, collected and placed in a new vial of the same capacity as the first, and corked. I investigated its composition at the same hour as that procured from the barrack, and found that it differed from it very sensibly. Its odor and its taste were almost neutral. It was without appreciable action upon test papers. I found in it spherical, ovoidal, and cylindrical bodies, but in quantities much less than in the first experiment.

But what I did not establish in this former, two hours after its condensation, was the existence of two bacteria catenula, formed of five joints, and of two wand-like vibriones performing their habitual movements, evidence that there may be found in the air living microzoa in a very advanced stage of development.

In the other examinations, made six hours and twenty-four hours after the condensation, I found the same diaphanous bodies, the same animalculæ, and the same spores as in that procured from the barrack, but in much less quantity.

The results of the microscopic examination were in harmony with those made by the unassisted senses, and with the more healthy condition of the casemate, by reason of its better ventilation.

EXPERIMENT III.

Made upon the external air to serve as a standard of comparison. Whilst I experimented in the barrack and in the casemate, my apparatus was also in operation at a point one metre above the highest portion of the fortification which overlookes the plain. The weather was fine, and the wind scarcely perceptible. I arranged that these three experiments should be made at the same time, in order that their results might be comparable and free from all objections.

I ought to mention that the chamber of the barrack, where I obtained so remarkable results, is at the same height as the fortification, and consequently, is supplied by the same stratum of air. The results which this experiment furnished are as follows: At the moment of the condensation, the liquid was limpid, colorless; its odor was that of the fresh, pure air which is inhaled in the open country in the morning. It was without action upon test papers. Its taste was fresh, and resembled that of pure water.

Examined with the microscope two hours after its condensation, I determined in it the existence of the spherical ovoid and cylindrical bodies before mentioned, but they were very scarce, and very small; of some fragments of dust and organic debris; finally, of cubical crystals, probably chloride of sodium, no animalculæ nor spores. Six hours after the condensation, I found the same things, but no animalculæ nor spores. Twenty-four hours after the condensation, I made, with the greatest care, six microscopic examinations, and found neither spores, nor bacteria, nor vibrions, nor monads. There were left only the diaphanous bodies referred to above. It was only after forty-eight hours after the condensation that I was able to establish the existence of some bacteria termo, of some very small wand-like vibriones, and of some very small spores, but no monads.

When these results are compared with those obtained in the two other experiments, one is struck with the difference which exists in the composition of the watery vapor collected in the open air, in the casemate, and especially in that of the chamber of the barrack.

At the end of six hours, there existed numerous animalculæ and spores in the confined air—I found them even two hours after the condensation, whilst a period of forty-eight hours was necessary to exhibit a few in the watery vapor collected in the open air.

This difference is maintained up to the end of the experiments, which I continued during ten days. I will narrate their details in a memoir.

Now, it remains for me to demonstrate what portions of the body furnish the microphytes and the microzoa referred to above, how they are disseminated into the air, and if they can be considered as the cause of typhus, of typhoid fever, and of other transmissible maladies. If the Academy will permit me, I will do myself the honor to discuss these questions at the next session.

